# The Importance of Trigeminal Cisternography in the Percutaneous Treatment of Idiopathic Trigeminal Neuralgia

**DOI:** 10.1155/prm/1625277

**Published:** 2025-09-27

**Authors:** Mustafa Balevi

**Affiliations:** Department of Neurosurgery, Konya Numune Hospital, Konya, Turkey

**Keywords:** glycerol, radiofrequency, trigeminal cisternography, trigeminal neuralgia

## Abstract

**Objective:** To investigate whether the tip of the spinal needle was accurately positioned within the trigeminal cistern (TC) in patients with idiopathic trigeminal neuralgia undergoing percutaneous treatments through the foramen ovale.

**Methods:** Transovale trigeminal cisternography (TOTC) was performed to ascertain the location of the spinal needle tip within the TC in 35 patients who underwent percutaneous retrogasserian glycerol rhizotomy (PRGR) procedures and 112 patients who underwent percutaneous controlled radiofrequency trigeminal rhizotomy (PCRF-TR) where the preganglionic trigeminal rootlets (PGTRs) could not be stimulated by radiofrequency. When TOTC revealed that the needle tip was in the subtemporal subarachnoid compartments, the needle insertion site at the foramen ovale was adjusted and redirected toward the dorsum sellae under intraoperative fluoroscopy guidance.

**Results:** In 17 (15%) patients who underwent PCRF-TR, PGTR stimulation was unsuccessful, and TOTC revealed the needle tip within the subtemporal subarachnoid space. In five (14%) patients who underwent PRGR, the needle tip was inside the subtemporal subarachnoid space, according to the TOTC evaluation.

**Conclusion:** The findings of this study underscore the significance of TOTC in both PRGR and PCRF-TR procedures, particularly when PGTRs cannot be stimulated by the radiofrequency electrode.

**Summary**

• The observation of CSF flow from the spinal needle after entering the FO percutaneously does not necessarily prove that the tip of the spinal needle is within the TC.
• CSF flow is observed when the spinal needle penetrates the subtemporal subarachnoid region within the MC. During PRGR, this complication can be easily diagnosed using TOTC.
• In PCRF-TR, if the preganglionic roots of the Gasserian ganglion (PGTR) cannot be stimulated, it is likely that the needle tip is located in the subtemporal subarachnoid region.

## 1. Introduction

Meckel's cave (MC) is a dural pouch containing cerebrospinal fluid (CSF) in the middle cranial fossa, opening from the posterior cranial fossa and housing the trigeminal ganglion. Located posterolaterally to the cavernous sinus on either side of the sphenoid bone, MC has the internal carotid artery medial to the Gasserian ganglion in the posterior portion of this sinus. Superiorly, the MC is bordered by the undersurface of the temporal lobe [1], and inferiorly, it lies adjacent to the motor root of the trigeminal nerve and the petrous apex, with the internal carotid artery traversing the carotid canal and sinuses [1, 2].

The trigeminal cistern (TC), a subarachnoid space within the posterior half of the MC, extends anteriorly along the trigeminal nerve rootlets and envelops the Gasserian ganglion to varying extents [2–4]. The anterior half of the MC, surrounded by the two layers of the dura mater and the pia mater, lacks CSF flow [3, 4].

Transovale TC (TOTC) utilizes a water-soluble contrast medium for the radiological examination of the TC [3, 4], TOTC was initially applied in 1911 for the examination of trigeminal neuralgia [5], and various techniques have since been developed to visualize the foramen ovale (FO) intraoperatively [6, 7]. Different original guide points have been utilized to visualize the FO using an intraoperative mobile C-arm X-ray machine. Grunert et al. proposed an alternative oblique X-ray trajectory for the correct placement of the needle into the FO on cadaveric skull models [8], while T. Gupta and S. K. Gupta reported the midline of the skull and the junction of the posterior wall and floor of the sellae as reliable landmarks for the identification of the FO [9].

TOTC, employing contrast agents, such as metrizamide or iohexol, is instrumental in visualizing the TC radiologically [10, 11]. Percutaneous treatments for idiopathic trigeminal neuralgia, including percutaneous retrogasserian glycerol rhizotomy (PRGR), percutaneous controlled radiofrequency thermocoagulation trigeminal rhizotomy (PCRF-TR), and balloon compression, are conducted through the FO [12–16]. While TOTC is utilized to assess TC volume during PRGR [10], there is a scarcity of studies in the literature examining its role in providing an understanding of the inability to stimulate preganglionic nerve roots during PCRF-TR. The current study aimed to investigate whether the tip of the spinal needle was accurately positioned within the TC in patients with idiopathic trigeminal neuralgia undergoing percutaneous treatments through the FO.

## 2. Methods

### 2.1. Patients

Between 2010 and 2020, a total of 147 patients with idiopathic trigeminal neuralgia were included in this study. Among them, 112 patients underwent PCRF-TR and 35 underwent PRGR. Ethical approval was obtained from the hospital's ethics committee (December 30, 2010: 78025658-641.99). Informed consent for the procedures performed was obtained from all participating patients. None of the patients had a history of a skull base fracture or surgical intervention related to the skull base.

Of the 147 patients included in the study, 63 were male and 84 were female. The mean age was found to be 49.3 ± 15.8 years. Among the 112 patients who underwent PCRF-TR, 50 were male and 62 were female, with mean ages of 56.5 ± 13.5 and 53.6 ± 16.5, respectively. Of the 35 patients who underwent PRGR, 13 were male and 12 were female, with mean ages of 53 ± 14.3 and 55.1 ± 15.2, respectively.

### 2.2. Percutaneous Trigeminal Cisternography Through the FO

Identification of FO by intraoperative fluoroscopy monitoring is an essential step for the correct placement of the cannula in the treatment of trigeminal neuralgia. The procedure began with the patient being placed in the supine position to visualize the FO. A mobile C-arm X-ray machine, providing sufficient image quality for clinical procedures, was used. The patient's head was extended, and the C-arm was tilted to provide an axial projection of the skull base, including the FO.

**Figure 1:**
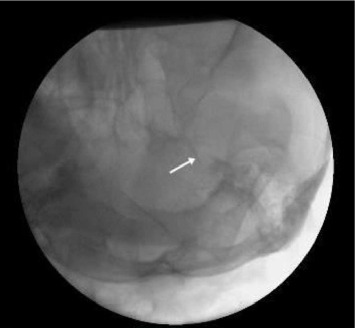
Submentovertical view of the skull showing the foramen ovale (white arrow).

The fluoroscopically guided needle insertion involved directing the spinal needle toward the medial ipsilateral pupillary line and 2 cm anterior to the external auditory canal. On the lateral view, the trajectory of the spinal needle was set 1.5 cm posterior to the dorsum sellae, not exceeding the clival line, before passing through the FO and entering the TC (Figure 1).

The topographic distribution of the trigeminal nerve at the FO includes the mandibular nerve laterally, the maxillary nerve in the midline, and the ophthalmic nerve medially. Under fluoroscopic guidance, the cannula was inserted into the desired part of the FO.

### 2.3. PRGR Procedure

**Figure 2:**
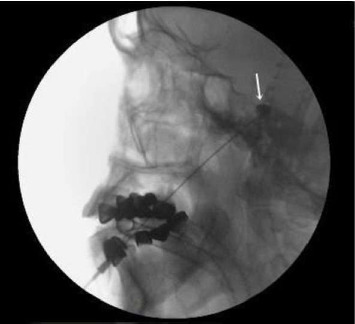
Lateral view of the skull showing the trigeminal cistern (white arrow).

**Figure 3:**
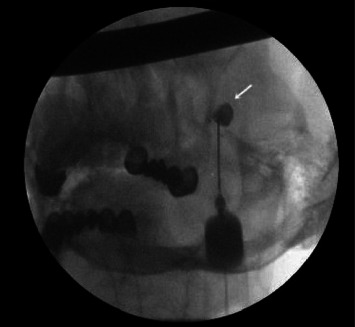
Anteroposterior view of the skull showing the trigeminal cistern (white arrow).

**Figure 4:**
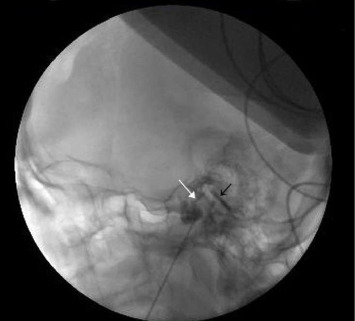
Lateral view of the skull showing the flow of contrast material from the porus trigeminus (white arrow) and the flow of contrast material into the posterior fossa (black arrow).

**Figure 5:**
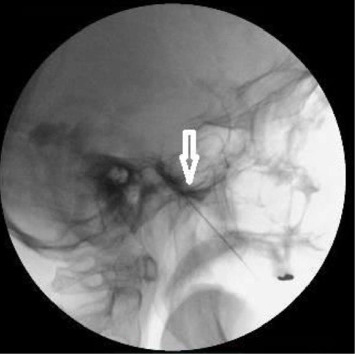
Lateral view of the skull showing the flow of contrast material into the subtemporal subarachnoid space (white arrow).

The patient was then placed in a sitting position, with an elevated and flexed head. A 0.2–0.5 mL contrast agent containing 647 mg of iohexol (equivalent to 300 mg of iodine per mL) was injected into the TC, visualized on both lateral (Figure 2) and anteroposterior (Figure 3) head radiographs. The TC volume was measured before the contrast medium passed into the posterior fossa (Figure 4). A glycerol dose of 0.3–0.5 cc was administered into the TC. If the tip of the spinal needle was visualized in the subtemporal subarachnoid space on TOTC (Figure 5), the entry point of the needle at the FO was adjusted, and the TC was punctured again. The contrast agent was injected again, and with the patient in the Trendelenburg position, the contrast agent was evacuated through the needle.

### 2.4. PCRF-TR Procedure

During PCRF-TR, the radiofrequency electrode was directed toward the preganglionic nerve roots, causing neuralgic pain. A low voltage was applied to stimulate these roots (Figure 1). If an electric-like paresthesia sensation could not be elicited in the area where neuralgic pain was experienced or in other trigeminal nerve regions, TOTC was performed.

The patient was placed in a sitting position, with the head elevated and flexed. A 0.2 mL contrast agent containing 647 mg of iohexol (equivalent to 300 mg of iodine per mL) was injected into the TC. The TC was visualized on both the lateral (Figure 2) and anteroposterior (Figure 3) head radiographs. If the tip of the spinal needle was observed in the subtemporal subarachnoid space on the lateral head radiograph (Figure 5), the entry point of the needle at the FO was adjusted. On the lateral view, the spinal needle trajectory was adjusted to be 1.5 cm posterior to the dorsum sellae, with a depth not exceeding the clival line. Subsequently, the radiofrequency electrode was used again to stimulate the preganglionic nerve roots. When an electric-like paresthesia sensation was obtained in the neuralgic pain area on the patient's face, thermocoagulation was applied to the preganglionic trigeminal root that caused this pain. The thermocoagulation process was typically applied at 60°C–70°C for 60–90 s, repeated three times depending on the affected branch.

### 2.5. Evaluation and Outcomes

The study evaluated the frequency of spinal needs tips being incorrectly positioned in the subtemporal subarachnoidal region during PRGR and PCRF-TR procedures. The incidence of this complication was compared between the two groups. In addition, patient complaints of headache, nausea, and vomiting due to iohexol were assessed, including the duration until symptom resolution.

### 2.6. Statistical Analysis

Data were analyzed using STATA Version 12 (Stata Corp., Texas, USA). The chi-square test and Student's *t*-test were used for statistical analysis. A *p* value of < 0.05 was considered statistically significant.

## 3. Results

In 124 (84%) of the 147 percutaneous procedures, CSF flow was observed from the spinal needle tip after entering the FO. In 23 cases (16%), CSF flow was not observed from the needle tip. TOTC revealed that in 22 (14.96%) cases, the spinal needle tip was located within the subtemporal subarachnoid space.

In 95 (85%) of the 112 patients who underwent PCRF-TR, stimulation applied with the parameters of 0.1 V, 50–60 Hz, and 3 ms duration, resulted in electric shock-like paresthesia in the trigeminal nerve areas where they experienced neuralgic pain. However, in the remaining 17 (15%) cases, despite stimulation with 0.5 V, 50–60 Hz, and 3 ms duration, paresthesia was not observed in the neuralgic pain area or other trigeminal nerve regions. In these cases, TOTC showed that the needle tip was within the subtemporal subarachnoid space.

In 30 (86%) of the 35 patients who underwent PRGR, the spinal needle tip was observed within the TC. In the remaining five (14%) cases, TOTC revealed that the spinal needle tip was within the subtemporal subarachnoid space.

Upon comparing the frequency of subtemporal subarachnoid puncture between the two percutaneous techniques, no significant difference was found (*p* > 0.05) (Table 1).

Iohexol injected intracisternally or subdurally caused a headache in 2% of the patients, which resolved approximately 2 h after the operation.

## 4. Discussion

The observation of CSF flow from the spinal needle after entering the FO percutaneously does not necessarily prove that the tip of the spinal needle is within the TC. CSF flow is observed when the spinal needle penetrates the subtemporal subarachnoid region within the MC. During PRGR, this complication can be easily diagnosed using TOTC. In PCRF-TR, if the preganglionic roots of the Gasserian ganglion (PGTR) cannot be stimulated, it is likely that the needle tip is located in the subtemporal subarachnoid region. This was also observed in cases where TOTC was performed in the current study.

There is a lack of literature regarding the use of TOTC in PCRF-TR. In the current study, TOTC was performed on 17 (15%) of the 112 PCRF-TR cases and five (14%) of the 35 PRGR cases, revealing that the needle tip was within the subtemporal subarachnoid space.

In this study, after the FO was visualized under fluoroscopic guidance, real-time fluoroscopic imaging was performed to perforate the FO using a spinal needle. This imaging facilitated the easy, quick, and successful negotiation of the nerve-block needle through the FO, as reported by Jeyaraj [17]. A modified fluoroscopically guided technique related to the transovale TC puncture was described by Gomori and Rappaport [7]. In another study, Lee et al. [18] reported that the mandible angle and the occipital cortex line were clear anatomical landmarks on seventy-two three-dimensional facial computed tomography (CT) scans without anatomical abnormalities of the skull. Schmidt et al. [19] used short sequence CT scans to visualize the FO. Bohnstedt et al. [20] noted that neuronavigation with intraoperative C-arm CT imaging was useful for visualizing the FO. Ding et al. [21] reported reaching the Gasserian ganglion through the FO using CT and neuronavigation from a mandibular angle.

Vascular complications associated with the needle trajectory can result in serious morbidity and mortality. The middle meningeal artery and distal branches of the maxillary artery are the most likely sources of percutaneous vascular injury [22]. In the Hartel technique, used to perforate the FO, injuries to the buccal nerve, facial nerve, or arteries may occur [23]. These complications were not observed in any of the patients included in the current study.

The relationship between the MC and the TC can vary depending on reference points on the skull base [24], making it difficult to puncture the TC via the FO with a spinal needle. Goerss et al. [25] reported that the appearance of the TC on TOTC can vary from person to person. There is a subdural extracisternal space within the MC where contrast material can be injected [1]. Puncturing the FO too laterally or too medially can cause the needle tip to reach the subtemporal subarachnoid space rather than the TC. Peris-Celda et al. [26] reported that knowledge of the extracranial and intracranial anatomical relationships of the FO was essential to understanding and avoiding complications during FO puncture and suggested that better radiographic visualization of the FO could improve lesioning accuracy depending on the part of the FO to be punctured. The angles and safety distances obtained may help neurosurgeons minimize complications during FO puncture and trigeminal neuralgia lesioning [26, 27].

CSF flow from the spinal needle tip was not observed in 23% of the patients included in this study. When the tip of the spinal needle is placed in the anterior part of the MC, contrast material cannot be injected into the MC, since there is no subarachnoid space in this region [1, 2]. Advancing the spinal needle a few millimeters forward in the MC can perforate the TC, resulting in CSF from the needle [28].

The volume of the TC is determined by TOTC. Glycerol should be administered into the cistern in an amount equal to the volume of the TC. Administering too much glycerol may lead to intracranial complications [28]. Some authors have reported that 0.15–0.2 mL of glycerol is sufficient for V3 and V2 branch trigeminal neuralgia, with even less needed for V1 neuralgia [13].

Intracisternal contrast agents may cause complaints such as headaches, nausea, and vomiting [29, 30]. Headache was observed in 2% of the patients who underwent TOTC in the current study. The dull headache caused by the contrast agent resolved 2 h after the operation.

The observation of CSF flow from the needle tip after entering the FO percutaneously does not prove that the needle tip is in the TC. When the spinal needle tip punctures the subtemporal subarachnoid region of the brain within the MC, CSF flow from the needle may be observed.

In cases where TOTC revealed incorrect needle placement and allowed for intraoperative repositioning, procedural efficacy appeared to improve. Although systematic recording of immediate clinical outcomes such as pain relief, complication rates, or the need for repeat interventions was not conducted, intraoperative observations indicated that repositioning the needle based on TOTC imaging enabled more precise targeting of the TC. This may have contributed to enhanced lesioning accuracy and reduced treatment failure. The absence of postprocedural complications beyond iohexol-related side effects in these cases further supports the clinical utility of TOTC as a corrective measure. Given these findings, the incorporation of TOTC into standard practice could serve not only as a diagnostic tool but also as a means to optimize clinical outcomes in real time. Future prospective studies are necessary to systematically evaluate these effects.

## 5. Conclusion

The findings of this study underscore the significance of TOTC in both PRGR and PCRF-TR procedures, particularly when PGTRs cannot be stimulated by the radiofrequency electrode.

## Figures and Tables

**Table 1:** Comparison of subtemporal subarachnoid puncture and trigeminal cistern puncture between the two percutaneous techniques.

	PRGR		PCRF-TR		*p* ^∗^
	*n*	%	*n*	%	
Subtemporal subarachnoid puncture	5	14	17	15	> 0.05
Trigeminal cistern puncture	30	86	95	85	> 0.05

Abbreviations: *n* = number, PCRF-TR = percutaneous controlled radiofrequency trigeminal rhizotomy, PRGR = percutaneous retrogasserian glycerol rhizotomy.

^∗^Statistically significant at *p* < 0.05.

## Data Availability

The data that support the findings of this study are available on request from the corresponding author. The data are not publicly available due to privacy or ethical restrictions.

## References

[1] Arslan M., Deda H., Avci E. (2012). Anatomy of Meckel’s Cave and the Trigeminal Ganglion: Anatomical Landmarks for a Safer Approach to Them. *Turkish Neurosurgery*.

[2] Sabancı P. A., Batay F., Civelek E. (2011). Meckel’s Cave. *World Neurosurgery*.

[3] Kaufman B., Bellon E. M. (1973). The Trigeminal Nerve Cistern. *Radiology*.

[4] Tsutsumi S., Ono H., Ishii H. (2021). Meckel Cave: An Anatomical Study Using Magnetic Resonance Imaging. *Journal of Computer Assisted Tomography*.

[5] Håkansson S. (1979). Transovale Trigeminal Cisternography. A Method for Radiological Examination of the Trigeminal Cistern Using a Water-Soluble Contrast Medium (Metrizamide). *Acta Neurochirurgia Supplementum*.

[6] Penman J. (1953). Some Developments in the Technique of Trigeminal Injection. *The Lancet*.

[7] Gomori J. M., Rappaport Z. H. (1985). Transovale Trigeminal Cistern Puncture: Modified Fluoroscopically Guided Technique. *AJNR: American Journal of Neuroradiology-NCBI*.

[8] Grunert P., Glaser M., Kockro R., Boor S., Oertel J. (2010). An Alternative Projection for Fluoroscopic-Guided Needle Insertion in the Foramen Ovale: Technical Note. *Acta Neurochirurgica*.

[9] Gupta T., Gupta S. K. (2012). Original Landmarks for Intraoperative Localization of the Foramen Ovale: A Radio-Anatomical Study. *Surgical and Radiologic Anatomy*.

[10] Van de Velde C., Smeets P., Caemaert J., Van de Velde E. (1989). Transoval Trigeminal Cisternography and Glycerol Injection in Trigeminal Neuralgia. *Journal Belge de Radiologie*.

[11] Christmann D., Babin E. (1980). Metrizamide Demonstration of the Subarachnoid Space Surrounding the Gasserian Ganglion. *Neuroradiology*.

[12] Arishima H., Kawajiri S., Arai H., Higashino Y., Kodera T., Kikuta K. (2016). Percutaneous Glycerol Rhizotomy for Trigeminal Neuralgia Using a Single-Plane, Flat Panel Detector Angiography System: Technical Note. *Neurologia Medico-Chirurgica*.

[13] Lunsford D. L., Bennett M. H. (1984). Percutaneous Retrogasserian Glycerol Rhizotomy for Tic Douloureux: Part 1. Technique and Results in 112 Patients. *Neurosurgery*.

[14] He Y., Zou C., Li Y. (2024). Balloon Pressure and Clinical Effectiveness of Percutaneous Microballoon Compression in the Treatment of Primary Trigeminal Neuralgia. *Pain Physician Journal*.

[15] Liu P., Zhong W., Liao C., Yang M., Zhang W. (2016). The Role of Percutaneous Radiofrequency Thermocoagulation for Persistent or Recurrent Trigeminal Neuralgia After Surgery. *Journal of Craniofacial Surgery*.

[16] Joswig H., Staudt M. D., MacDougall K. W., Parrent A. G. (2020). Effect of Training on Percutaneous Glycerol Rhizotomy for Trigeminal Neuralgia: A Long-Term, Retrospective Comparison of Staff Neurosurgeon and Trainee Complications and Efficacy. *World Neurosurgery*.

[17] Jeyaraj P. (2022). Efficiency and Efficacy of Real-Time Fluoroscopic Image-Guided Percutaneous Gasserian Glycerol Rhizotomy (PGGR), for Intractable Cases of Trigeminal Neuralgia. *Journal of Maxillofacial and Oral Surgery*.

[18] Lee S. H., Kim K. S., Lee S. C. (2019). A Novel Method of Locating Foramen Ovale for Percutaneous Approaches to the Trigeminal Ganglion. *Pain Physician*.

[19] Schmidt B. T., Pun C. D., Lake W. B., Resnick D. K. (2020). Computed Tomography Guidance for Percutaneous Glycerol Rhizotomy for Trigeminal Neuralgia. *Operative Neurosurgery*.

[20] Bohnstedt B. N., Tubbs R. S., Cohen-Gadol A. A. (2012). The Use of Intraoperative Navigation for Percutaneous Procedures at the Skull Base Including a Difficult-to-Access Foramen Ovale. *Operative Neurosurgery*.

[21] Ding W., Chen S., Wang R. (2016). Percutaneous Radiofrequency Thermocoagulation for Trigeminal Neuralgia Using Neuronavigation-Guided Puncture From a Mandibular Angle. *Medicine (Baltimore)*.

[22] Lawrence J. D., Cheyuo C., Marsh R. A. (2022). Infratemporal Fossa Vascular Anatomy Pertinent to Percutaneous Access to the Foramen Ovale for Treatment of Trigeminal Neuralgia: A Comparison of Cadaveric Dissection and Computed Tomography Analysis. *World Neurosurgery*.

[23] Iwanaga J., Badaloni F., Laws T., Oskouian R. J., Tubbs R. S. (2018). Anatomic Study of Extracranial Needle Trajectory Using Hartel Technique for Percutaneous Treatment of Trigeminal Neuralgia. *World Neurosurgery*.

[24] Liu H. B., Ma Y., Zou J. J., Li X. G. (2007). Percutaneous Microballoon Compression for Trigeminal Neuralgia. *Chinese Medical Journal*.

[25] Goerss S. J., Atkinson J. L., Kallmes D. F. (2009). Variable Size Percutaneous Balloon Compression of the Gasserian Ganglion for Trigeminal Neuralgia. *Surgical Neurology*.

[26] Peris-Celda M., Graziano F., Russo V., Mericle R. A., Ulm A. J. (2013). Foramen Ovale Puncture, Lesioning Accuracy, and Avoiding Complications: Microsurgical Anatomy Study With Clinical Implications. *Journal of Neurosurgery*.

[27] Liu H., Xu L., Zhao W. (2023). Puncture Approaches and Guidance Techniques of Radiofrequency Thermocoagulation Through Foramen Ovale for Primary Trigeminal Neuralgia: Systematic Review and Meta-Analysis. *Frontiers in Surgery*.

[28] Håkanson S. (1981). Trigeminal Neuralgia Treated by the Injection of Glycerol Into the Trigeminal Cistern. *Neurosurgery*.

[29] Skalpe I. O. (1977). Adverse Effects of Water-Soluble Contrast Media in Myelography, Cisternography and Ventriculography. A Review With Special Reference to Metrizamide. *Acta Radiologica-Supplement*.

[30] Wang Y. S., Jiang Y. H., Hou Z. Y. (1990). Intrathecal Injection of Iohexol for Routine Myelography and CT Myelography in 1000 Cases. *Chinese Medical Journal*.

